# Clinical teaching self-efficacy positively predicts professional fulfillment and negatively predicts burnout amongst Thai physicians: a cross-sectional survey

**DOI:** 10.1186/s12909-024-05325-1

**Published:** 2024-04-02

**Authors:** Arunee Tipwong, Nathan C. Hall, Linda Snell, Parinya Chamnan, Matthew Moreno, Jason M. Harley

**Affiliations:** 1 Department of Educational and Counselling Psychology, McGill University, Montreal, QC Canada; 2 Institute of Health Sciences Education, McGill University, Montreal, QC Canada; 3 Division of General Internal Medicine, Faculty of Medicine, McGill University, Montreal, QC Canada; 4 Department of Surgery, Faculty of Medicine, McGill University, Montreal, QC Canada; 5 Research Institute of the McGill University Health Centre, Montreal, QC Canada; 6 Department of Social Medicine, Surat Thani Hospital, Surat Thani, SNI Thailand; 7 Office of the Collaborative Project to Increase Production of Rural Doctor (CPIRD), Ministry of Public Health, Nonthaburi, NBI Thailand

**Keywords:** Burnout, Fulfillment, Quality of life, Quantitative, Retention, Teacher self-efficacy

## Abstract

**Background:**

Clinician teachers (physicians who teach in clinical settings) experience considerable psychological challenges in providing both educational training and patient care. This study aimed to determine the prevalence of physician burnout and professional fulfillment, and to identify internal and external factors associated with mental health outcomes among Thai clinician teachers working in non-university teaching hospitals.

**Method:**

A one-time online questionnaire was completed by physicians at 37 governmental, non-university teaching hospitals in Thailand, with 227 respondents being assessed in the main analyses. Four outcomes were evaluated including burnout, professional fulfillment, quality of life, and intentions to quit.

**Results:**

The observed prevalence of professional fulfillment was 20%, and burnout was 30.7%. Hierarchical regression analysis showed a significant internal, psychological predictor (clinical teaching self-efficacy) and external, structural predictors (multiple roles at work, teaching support), controlling for the background variables of gender, years of teaching experience, family roles, and active chronic disease, with clinical teaching self-efficacy positively predicting professional fulfillment (*b* = 0.29, *p* ≤.001) and negatively predicting burnout (*b* = − 0.21, *p* =.003).

**Conclusions:**

Results highlight the importance of faculty development initiatives to enhance clinical teaching self-efficacy and promote mental health among Thai physicians.

**Supplementary Information:**

The online version contains supplementary material available at 10.1186/s12909-024-05325-1.

## Background

### Why is physician mental health of concern?

Physician mental health has been recognized as a significant issue in the healthcare system due to its various negative impacts on physicians and patients. Studies over the past decades have found that physicians with mental health issues can jeopardize patient care quality and safety [1–6]. Awareness and promotion of physician mental well-being are, thus, essential to an efficient healthcare system. Physician burnout is an occupational hazard in medical settings, with burnout being defined as resulting from chronic workplace stress and characterized by emotional exhaustion, cynicism or depersonalization, and feelings of reduced personal accomplishment. For example, occupational burnout has been found to contribute to major depressive disorder, sleep disturbance, chronic myofascial pain syndrome, metabolic syndrome, type 2 diabetes, hypertension, cardiovascular diseases, inflammatory problems, immunity problems, as well as cancer and gastrointestinal problems [7]. Therefore, it is essential to evaluate and prevent burnout in physicians, and to further explore how other roles in addition to being a care provider, such as teacher, administrator, or researcher, may further contribute to work stress and ill health.

Global studies have shown physician burnout to vary in prevalence from 22.2 to 99.6% [2, 8–19]. Studies in primary care settings, in particular, have found the prevalence of burnout in physicians to vary widely based on not only burnout definition but also workplace factors (e.g., from 19 to 81% [20–22].

### Factors associated with physician mental health

Studies have shown various factors to be related to physician mental health, with these influences typically categorized into internal and external factors. Internal factors represent all aspects of an individual, such as gender, age, skills, personality, and perceptions. Many studies have found that female physicians report more mental health issues such as burnout than do male physicians [3, 17, 23–26] with work-home conflict proposed as a possible reason for this gender difference [3, 24, 27]. However, some studies have found no significant difference in burnout between male and female physicians [2, 5, 11, 26]. Findings suggest that older age and/or greater years of experience may be a preventive factor for physician burnout [3, 4, 17, 28]. Physician burnout has also been found to be associated with psychological factors such as specific psychological characteristics, emotional intelligence, and work attitude [17, 26, 29]. Personal health and lifestyle issues such as sleep disturbance, pain, low frequency of exercise, and high alcohol consumption have also been found to negatively impact physician burnout [11, 30]. Other studies have similarly found that self-reported poor sleep/rest quality are significantly associated with higher physician burnout [8, 31].

External factors have indicated that physicians who indicated marital status as single have been found to report higher burnout, with physicians who indicated being married (i.e., having a supportive spouse) or having children reporting lower burnout levels [16, 17]. Lack of autonomy at work has been found to correspond with higher physician burnout in multiple studies [3, 4, 27, 29]. Physicians with greater authority or leadership positions have been found to report lower burnout and greater fulfillment [23, 32], with academic physicians who experienced conflicts with hospital leaders also reporting higher burnout [4].

Similarly, many studies reported that high workloads and long work hours are linked to high burnout or poor quality of life in physicians [4, 5, 11, 27, 29, 33]. Physicians who perceive their work demands as infringing on their home life have also been shown to report greater burnout [4, 27, 29]. Moreover, work-related pressure has been found to indirectly predict stronger intentions to quit the medical profession among physicians due to the mediational role of greater burnout [34].

### Clinical instruction and mental health

Physicians who teach in clinical settings, known as clinician teachers, thus typically have more roles and responsibilities than physicians working primarily as clinicians. Clinician teachers must thus contend with the work-related stress of maintaining up-to-date medical knowledge, care delivery, and teaching capabilities, while also faced with high workload and often limited support or resources [35].

Considering the added complexity and responsibility of clinical teaching, it is reasonable to expect that physicians’ internal perceptions of incompetence or low self-efficacy could significantly impact their well-being, particularly for novice clinician teachers. Similarly, although findings on the psychological demands of taking on additional teaching roles as a physician are mixed [6, 11, 36–38] published research on this topic is limited and may not adequately reflect current trends in medical education. Relatedly, whereas existing research on clinician teachers suggests that exploring internal and external factors may be useful for understanding the experiences of physicians who teach, this topic has yet to be examined in empirical research with Thai clinician teachers.

### Physician mental health in Thailand

Although physician mental health has been studied worldwide, there are few studies conducted in the Thai context. Sithisarankul and colleagues surveyed the mental health of 440 Thai physicians using the Thai General Health Questionnaire (Thai GHQ-28) and found that physicians who reported abnormal mental health status (7.4%) also reported significantly lower career satisfaction [39]. Related study findings nevertheless showed high-overall satisfaction levels among Thai physicians (60.2%) [40, 41]. However, findings from another survey conducted in 2004 with 327 Thai physicians showed 20% to have chronic diseases such as hypertension, diabetes mellitus, and ischemic heart disease, for which adequate treatment depends on lifestyle modifications [42]. A cross-sectional study [8] with 245 Thai physicians working in secondary and tertiary care hospitals in the southernmost region of the country further compared the prevalence of burnout between physicians in regions with low vs. high levels of political conflict and unrest using the Maslach Burnout Inventory (Thai version).

In summary, although studies of physician mental health in Thailand show considerable variability on measures of quality of life, only one study to date has examined physician burnout and none have focused specifically on the role of clinical teachers. Accordingly, there is a need for further research on the prevalence of Thai physician burnout across the country and the factors associated with greater burnout, particularly in teaching hospitals in the public health sector where physicians have additional teaching roles alongside their other fundamental roles as physicians in governmental non-teaching hospitals.

In the present cross-sectional study, we addressed these gaps in the literature with two main objectives: (1) identify the prevalence of burnout and professional fulfillment in Thai physicians who teach in Medical Educational Centres (MECs); and (2) determine critical internal and external factors related to mental health in Thai physicians as assessed with both positive and negative indicators including burnout, professional fulfillment, quality of life, and intentions to quit.

## Methods

### Participants

Clinician teachers were recruited from all 37 MECs across Thailand. Inclusion criteria included participants being active clinical teachers at an MEC during their time in the study; no exclusion criteria beyond this were employed. A convenience sampling technique helped to capture the widest cross section of medical physicians. A recruitment letter and the link to the online questionnaire were distributed by the directors and educational officers at each MEC. Data was collected from April through June 2021. Participants included 297 physicians; 70 were excluded from further analysis due to missing data, resulting in 227 participants being assessed in the main analyses. A retrospective power analysis was conducted using G*Power version 3.1.9.7 [43] to calculate the sample size needed in this study. In our calculations, we used a smaller effect size (0.3) using Cohen’s [44] criteria that was more conservative in nature. In step 1 of our calculations, with a sample of two-hundred and twenty-seven participants (*N* = 227), 4 predictors, and our sample and effect sizes, we achieved a nominal power of 0.99. In step 2 of our calculations, we increased to 7 predictors, and our sample size and effect size, to achieve a nominal power of 0.99. The response rate was 9%, with the sample size considered sufficient considering power analyses (*n4studies* application) based on previously published studies from Ganeshan et al. [4] (infinite population proportion, *p* =.79, error = 0.035, power = 0.80, alpha = 0.05), and Pitanupong and Jatchavala [8] (infinite population proportion, *p* =.99, error = 0.035, power = 0.80, alpha = 0.05).This study received institutional research ethics approval and was designated low risk to human subjects.

### Study measures

The present study focused on both positive and negative indicators of mental health in Thai physicians, collecting data using the following tools:

1. The *Professional Fulfillment Index (PFI)* was developed by Trockel et al. [45] to assess mental health among physicians. The scale contains three subscales assessed on a five-point Likert scale: professional fulfillment (six items), work exhaustion (four items) and interpersonal disengagement (six items). Overall burnout was calculated by combining the work exhaustion and interpersonal disengagement subscales. Construct validity of the PFI has been confirmed by Trockel and colleagues [45] who report strong Cronbach’s alpha coefficients for professional fulfillment (0.91) and overall burnout (0.92), and high test-retest reliability for professional fulfillment (0.82) and overall burnout (0.80). The authors also provided the ROC analysis for a cut-off point to determine professional fulfillment at a mean score of 3, with values at or above this score reflecting “very good” quality of life (sensitivity: 0.73, specificity: 0.79). The cut-off point for burnout was established in ROC analyses by the authors as scores higher than 1.33 based on three comparison scales including the MBI (sensitivity: 0.72 − 0.85, specificity: 0.76 − 0.84).
2. *EURO Quality of Life Five-Dimension–Five-Level Scale (EQ5D5L)* was developed by the EuroQOL group [46] assesses perceived quality of life along five dimensions including mobility, self-care, usual activities (e.g., work, study, housework, leisure activities), pain/discomfort, and anxiety/depression (scale preamble: “How challenging of each of these health issues for you TODAY?”). Each dimension is then assessed by respondents according to five response levels (no, slight, moderate, severe, extreme). Accordingly, higher scores on this measure indicate *poorer* quality of life. A Thai-language version of this scales has been previously validated [47].
3. As there is currently no scale developed specifically to measure clinical teaching self-efficacy in medical education contexts, a modified version of the Maastricht Clinical Teacher Questionnaire (MCTQ) was administered to assess physicians’ self-efficacy pertaining to clinical teaching. The 14-item, five-point MCTQ [48] assessed clinical teachers’ perceived ability to coach learners, articulate concepts, explore themes, and provide meaningful feedback (1 = *strongly disagree*, 5 = *strongly agree.* The MCTQ has been validated to use as an instrument for the evaluation of clinical teachers [49].
4. Perceived teaching support was assessed using a single item with three response options from without support to fully supported. Teaching experience was measured as the number of years since participants started their teaching roles.
5. Intentions to quit was assessed using a scale adapted from a work commitment scale by Hackett et al. [50] to reflect the work conditions of governmental-employed physicians. This scale consisted of three items assessed on a five-point scale (1 = strongly disagree, 5 = strongly agree) including “I have considered quitting one or more of my professional roles (e.g., teaching, administration),” “I have considered leaving public practice for private practice,” and “I have considered leaving the medical profession for another profession.”

Cronbach’s alpha coefficients were calculated by the authors of the present study and deemed to be satisfactory for all self-report, multi-item scales including teacher self-efficacy (0.91), fulfillment (0.85), quality of life (0.82), burnout (0.90), and quitting intentions (0.71) [Table 1in Supplemental Digital Content].

## Results

Most participants were female (67.7%) with a mean age of 42.81 years (*SD* = 7.71). This proportion is similar to the statistics reported from the Thai Medical Council that the number of female physicians was greater than males in the age group of 31 to 50 years old [51].

The prevalence of professional fulfilment and burnout as determined by the cut-off thresholds were 20% for fulfillment and 30.7% for burnout. The main hierarchical linear regression analyses on the four mental health outcomes are shown in Table 1. In Step 1, the following variables were assessed: Gender (Female), years of teaching experience, multiple family roles, and chronic disease. Results showed *R*^*2*^ for all outcomes in the first step to be statistically significant, with the highest proportion of variance explained for quality of life (*R*^*2*^ = 0.10, *p* ≤.001).

**Table 1:** Hierarchical Linear Regressions on Clinician Teachers’ Mental Health

Variables	Fulfillment	Quality of life^†^	Burnout	Quitting
	β	β	β	β
**Step 1**				
Gender (Female)	0.110	0.063	− 0.156*	− 0.064
Years of teaching experience	0.166*	− 0.030	− 0.126	− 0.091
Multiple family roles	0.050	0.230**	0.056	0.030
Chronic disease	− 0.188**	0.182**	0.161*	0.201**
*R*^*2*^	0.064**	0.100***	0.061*	0.051*
**Step 2**				
Gender (Female)	0.119	0.059	− 0.163*	− 0.063
Years of teaching experience	0.144*	− 0.015	− 0.121	− 0.069
Multiple family roles	− 0.004	0.250***	0.088	0.050
Chronic disease	− 0.213**	0.189**	0.173*	0.213**
Clinical teaching self-efficacy	0.292***	− 0.102	− 0.206**	− 0.051
Multiple work roles	0.100	− 0.031	− 0.022	− 0.104
Organizational teaching support	0.066	0.055	− 0.057	0.024
*R*^*2*^	0.165***	0.115	0.106*	0.066
Δ*R*^*2*^	0.101***	0.016	0.044*	0.015

^†^ Higher score refers to poorer quality of life in Euro Quality of life scale (EQ5D5L); * *p* <.05. ** *p* <.01. ***p <.001

In Step 2 of the regression, the following variables were additionally included: clinical teaching self-efficacy, multiple work roles, and organizational teaching support. *R*^*2*^ changes were statistically significant for professional fulfillment (D*R*^*2*^ = 0.101, *p <*.001) and burnout (D*R*^*2*^ = 0.044, *p* =.022). Of the three predictor variables added in Step 2, only clinical teaching self-efficacy significantly predicted mental health outcomes. Specifically, higher teaching self-efficacy had a positive and statistically significant effect on predicting professional fulfillment (*b* = 0.29, *p* ≤.001) and a negative and statistically significant effect on predicting burnout (*b* = − 0.21, *p* =.003). Results showed that clinical teaching self-efficacy positively predicted professional fulfillment after controlling for chronic disease [see also Table 2; Fig. 1 in Supplemental Digital Content].

## Discussion

The present research focused on the experiences of physicians employed as clinician teachers in public-service hospitals in Thailand. To address this research gap, the study assessed the prevalence of burnout and fulfillment in clinician teachers in Thai MEC contexts, and to what extent both internal, psychological factors (teaching self-efficacy) and external, structural factors (multiple work roles, teaching support) affected their mental health outcomes with respect to burnout, fulfillment, quality of life, and intentions to quit.

Using the Professional Fulfillment Index (PFI), results showed physician burnout to be 30.7%, similar to those reported by current reports [31, 38, 52] amongst academic physicians. These findings are also notable because similar levels of burnout were also observed using the MBI (Maslach Burnout Inventory) thus corroborating the use of the PFI scale to determine burnout in previous work [52]. The burnout prevalence found in the present study differs considerably from that previously reported among Thai physicians [8], with this discrepancy likely the result of differences in the burnout scales assessed and cut-off points used to classify burnout. Whereas prior studies have used the MBI and classified burnout as any total score higher than zero, the present study used the PFI and classified burnout more conservatively as total averaged scores of 1.33 or higher; a cut-off value identified by an ROC analysis [45].

Results describing the prevalence of positive physician wellbeing showed the prevalence of professional fulfillment to be 20%, which is lower than recent findings with the PFI (35.6%) [53]. Moreover, this positive work-related mental health outcome found in the present study, is less prevalent than burnout. This could suggest that mental health among Thai physicians remains a cause for concern, particularly when they are being burdened with additional teaching responsibilities. This also highlights the importance of using positively valenced scales to assess physician mental health in Thailand, as although results of negative indicators may be consistent with other populations, results of positive indicators may help to provide a more complete perspective on how Thai physicians are coping with teaching demands.

The main findings reiterated the importance of examining both internal and external factors that may predict Thai clinician teachers’ mental health outcomes after controlling for critical background variables (i.e., gender, years of teaching experience, number of family roles, and chronic disease). Although clinical teaching self-efficacy predicted higher levels of professional fulfillment and lower burnout, it did not significantly predict their quality of life or quitting intentions. The relationship between teacher self-efficacy and burnout is thus similar to previous findings with K-12 teachers [36] and with non-medical university faculty [54] and underscores the importance of clinical teaching self-efficacy as a critical internal psychological resource for clinician teachers.

According to social learning theory [55], self-efficacy is defined as beliefs concerning one’s personal capacity to effectively perform specific actions in a given achievement setting, with these beliefs further proposed to correspond with subsequent behaviours and environmental outcomes. As such, the present findings suggest that when a clinician teacher perceives low self-efficacy in teaching, they are more likely to teach more poorly and receive more negative feedback from students and impair well-being. On the other hand, when the clinician teacher perceives greater self-efficacy, the anticipated self-confidence they convey while teaching and positive feedback from students is expected to positively contribute to their well-being. According to Kunter et al., [56], greater teaching self-efficacy should also indirectly impact student outcomes such as learning-related motivation and enjoyment. Hence, this finding showcases the importance of supporting and promoting teacher self-efficacy in Thai MEC settings considering the potential benefits for teaching effectiveness, student learning, and the well-being of both clinician teachers and students.

Although previous literature reports mixed effects of multiple roles at work and teaching support on physician burnout and professional fulfillment [6, 37, 57, 58], our findings showed both multiple roles and organizational teaching support to not significantly correspond with any of the four mental health outcomes assessed. A possible reason for these findings may involve the specific context of the work environment and the organizational system experienced by the study participants. It is possible that study participants may had already developed sufficient resilience to the stress of their workload, and were less impacted by multiple roles, than were Thai physicians in less demanding environments. Concerning the lack of results for teaching support, it is possible that teaching resources were not yet sufficient to match the teaching demands of clinician teachers (e.g., workshops, materials) resulting in limited scale variability and a consequent lack of relations.

With respect to study limitations, whereas an online survey was selected to recruit the largest cross section of participants possible, it is possible that participants were more likely to be digital natives thereby excluding older, non-digital participants. In addition, as all study measures were self-report in nature, future studies should aim to include qualitative data (e.g., focus groups, in-depth interview) to provide a more in-depth perspective. As the present study was conducted during COVID-19, it is possible that challenges specific to the pandemic may have negatively affected the mental health of the participants [59], availability of teaching supports, or physicians’ willingness to participate in the study (i.e., contributing to reduced power to detect effects).

Finally, it should be noted that although there exists ongoing conflict in southern Thailand, the present study was not recently preceded by a major conflict-related event and the research questions were not specific to southern Thailand [8]. Instead, the present study addressed physician responses to a nation-wide pedagogical initiative across both northern and southern Thailand. Accordingly, conflict-related issues were considered beyond the scope of the present research questions and were not addressed in the self-report survey distributed to physicians in this study.

## Conclusions

The present study aimed to identify the prevalence of burnout and professional fulfillment, as well as how internal and external predictive factors correspond with varied mental health outcomes for Thai physicians who have in recent years been required teach as per a government initiative to improve physician education across the country. Study findings showed a burnout prevalence of 30.7% equivalent to prior research with academic physicians internationally, however the observed professional fulfillment prevalence of 20% indicated that Thai physician well-being in MEC settings remains a cause for concern. Teaching self-efficacy was also found to be a significant predictor of lower burnout and higher professional fulfillment in Thai clinician teachers controlling for gender, years of teaching experience, family roles, and chronic disease. Taken together, all these indicate the importance of internal psychological factors for promoting well-being in clinical education. It is expected that faculty development programs that enhance teaching self-efficacy could help to improve professional fulfillment and reduce burnout among physicians tasked with clinical teaching at public-service hospitals in Thailand.

### Electronic supplementary material

Below is the link to the electronic supplementary material.

Supplementary Material 1

## Data Availability

The datasets generated and analysed during the current study are not publicly available due ethical constraints but are available from the corresponding author on reasonable request.
