# Correction: Clinical teaching self-efficacy positively predicts professional fulfillment and negatively predicts burnout amongst Thai physicians: a cross-sectional survey

**DOI:** 10.1186/s12909-024-05483-2

**Published:** 2024-05-10

**Authors:** Arunee Tipwong, Nathan C. Hall, Linda Snell, Parinya Chamnan, Matthew Moreno, Jason M. Harley

**Affiliations:** 1 Department of Educational and Counselling Psychology, McGill University, Montreal, QC, Canada; 2 Institute of Health Sciences Education, McGill University, Montreal, QC, Canada; 3 Division of General Internal Medicine, Faculty of Medicine, McGill University, Montreal, QC, Canada; 4 Department of Surgery, Faculty of Medicine, McGill University, Montreal, QC, Canada; 5 Research Institute of the McGill University Health Centre, Montreal, QC, Canada; 6 Department of Social Medicine, Surat Thani Hospital, Surat Thani, SNI, Thailand; 7 Office of the Collaborative Project to Increase Production of Rural Doctor (CPIRD), Ministry of Public Health, Nonthaburi, NBI, Thailand

**Correction: BMC Medical Education (2024) 24:361**

10.1186/s12909-024-05325-1

Following publication of the original article [1], the authors reported that there is a misspelling of a measure listed within the manuscript.

Incorrect: EURO Quality of Life Five-Dimension–Five-Level Scale (Eq. 5D5L)

Correct: EURO Quality of Life Five-Dimension–Five-Level Scale (EQ5D5L)

The original article has been corrected.

## References

[1] Tipwong et al. BMC Medical Education (2024) 24:361 10.1186/s12909-024-05325-1. 10.1186/s12909-024-05325-1 PMC10988928 38566108

