# Supplementary material for: Clinical teaching self-efficacy positively predicts professional fulfillment and negatively predicts burnout amongst Thai physicians: a cross-sectional survey
Source: BMC Med Educ. 2024 Apr 2;24:361. doi: 10.1186/s12909-024-05325-1 (PMC10988928; doi:10.1186/s12909-024-05325-1)
Supplement: Supplementary file 1 — Supplementary Material 1 [file 12909_2024_5325_MOESM1_ESM.docx]

**Supplemental Digital Content**

Table 1. Descriptive Statistics for Study Measures

|  | *α* | *M* | *SD* | *n* | No. of items | Actual range |
| --- | --- | --- | --- | --- | --- | --- |
| Teacher self-efficacy | .91 | 53.98 | 6.66 | 203 | 14 | 1-5 |
| Fulfillment | .85 | 15.18 | 4.22 | 212 | 6 | 0-4 |
| Quality of life | .82 | 7.79 | 2.74 | 208 | 5 | 1-5 |
| Burnout | .90 | 10.98 | 6.57 | 208 | 10 | 0-4 |
| Quitting intentions | .71 | 6.61 | 2.65 | 211 | 3 | 1-5 |

Figure 1. Hierarchical Regression Model

Table 2. Zero-order Correlations among Study Variables

| Variables | 1 | 2 | 3 | 4 | 5 | 6 | 7 | 8 |
| --- | --- | --- | --- | --- | --- | --- | --- | --- |
| 1. Teaching experience | - |  |  |  |  |  |  |  |
| 2. Family roles | .222*** | - |  |  |  |  |  |  |
| 3. Teaching self-efficacy | .077 | .173* | - |  |  |  |  |  |
| 4. Work roles | .257*** | .147* | .182** | - |  |  |  |  |
| 5. Teaching support | -.106 | -.017 | -.078 | -.052 | - |  |  |  |
| 6. Fulfillment | .147* | .032 | .287*** | .126 | .000 | - |  |  |
| 7. Quality of life^†^ | -.030 | .210** | -.058 | .018 | .084 | -.294*** | - |  |
| 8. Burnout | -.085 | .066 | -.171* | -.016 | .005 | -.544** | .306*** | - |
| 9. Quitting | -.084 | .039 | -.047 | -.059 | .065 | -.473*** | .346*** | .414*** |

^†^ Higher EQ5D5L scores indicate poorer quality of life. **p* < .05. ***p* < .01. ****p* < .001
